# Examining park features that encourage physical activity and social interaction among adults

**DOI:** 10.1093/heapro/daaf063

**Published:** 2025-06-02

**Authors:** Jenny Veitch, Elise Rivera, Venurs Loh, Chahana Paudel, Nicole Biggs, Benedicte Deforche, Anna Timperio

**Affiliations:** School of Exercise and Nutrition Sciences, Deakin University, Geelong, Australia; Institute for Physical Activity and Nutrition (IPAN), 221 Burwood Highway, Burwood, 3125 Victoria, Australia; School of Health, Medical, and Applied Sciences, Appleton Institute, Central Queensland University, 44 B26, Wayville, South Australia 5034, Australia; College of Sport, Health, and Engineering, Victoria University and Institute for Health and Sport, Victoria University, 70–104 Ballarat Road, Footscray, 3011 Victoria, Australia; Institute for Health and Sport, Victoria University, 70–104 Ballarat Road, Footscray, 3011 Victoria, Australia; School of Exercise and Nutrition Sciences, Deakin University, Geelong, Australia; Murdoch Children’s Research Institute, 50 Flemington Road Parkville, 3052 Victoria, Australia; Faculty of Medicine and Health Sciences, Department of Public Health and Primary Care, Ghent University, C. Heymanslaan 10, 9000 Ghent, Belgium; Movement and Nutrition for Health and Performance Research Group, Faculty of Physical Education and Physical Therapy, Department of Movement and Sport Sciences, Vrije Universiteit Brussel, Pleinlaan 2, 1050 Brussels, Belgium; School of Exercise and Nutrition Sciences, Deakin University, Geelong, Australia

**Keywords:** green space, healthy cities, urban parks, design

## Abstract

Urban parks are a critical setting that can support good health by providing opportunities for physical activity and social interaction. Therefore, it is necessary to better understand which park features are most important for encouraging active park use and social interaction. This study examined the perceived importance of park features for encouraging park visitation, park-based physical activity, and social interaction among adults in Australia. Participants [*n* = 232, 42.2 years (SD = 13.4), 53% female] rated images of 43 different park features. For each feature, mean scores (SD) were calculated for the overall sample and by gender and frequent/infrequent park visitors for each park-use behaviour (visit, active, social). ‘Good maintenance and cleanliness’ and ‘trees’ were the two highest-rated features for encouraging adults to visit a park and be active and social. A ‘peaceful and relaxed setting’ was ranked third for visitation and ‘sense of safety from strangers’ was ranked third for both physical activity and social interaction. Significant differences in rating scores were observed between frequent and infrequent park visitors for all three outcomes. The findings will assist those involved in park planning and design to create parks that are tailored to the specific needs of this important user group. This is critical for enhanced physical, social, and mental health at the population level.

Contribution to Health Promotion

- This study provides insights into the most important park features for encouraging active park use and social interaction among adults.
- Good maintenance and cleanliness, trees, a peaceful and relaxed setting, and a sense of safety from strangers were the highest-rated park features.
- Findings will inform future park planning and design to encourage adults to engage in physical activity and social interaction in parks, which will support physical, social, and mental health.

## BACKGROUND

High-level policy frameworks and plans, such as the United Nation’s 2030 Agenda for Sustainable Development (United Nations 2015), World Health Organization’s Global Action Plan on Physical Activity 2018–2030 (World Health Organization 2018), and the United Nation’s New Urban Agenda, Habitat III (United Nations General Assembly 2016), recognize the importance of creating cities and active environments, such as urban green spaces, that enhance health and urban liveability. Urban neighbourhood parks are important public settings that can foster many physical, mental, and social benefits by providing opportunities for physical activity, social interaction, and connection with nature (Douglas *et al*. 2017).

The provision of high-quality urban parks that support active park use may help combat low population physical activity levels by facilitating active recreation within the park. They can also support active transport as parks are often reachable on foot or by bike in urban areas (Koohsari *et al*. 2015). This is important as 28% of adults worldwide were insufficiently active in 2016 in a pooled analysis of 358 population-based surveys (Guthold *et al*. 2018), and approximately 55% of Australian adults did not adhere to Australian Physical Activity Guidelines from 2017 to 2018 (Commonwealth of Australia 2021).

There is significant potential to harness parks to confer multiple health benefits for the adult population by increasing park visitation and enhancing physical activity (Joseph and Maddock 2016). A review of observational studies in parks suggested that adults were usually observed being less active than youth (Evenson *et al*. 2016), and fewer females than males were observed engaging in park-based physical activity (Joseph and Maddock 2016). Females have also reported less frequent and shorter park visits compared to males (Derose *et al*. 2018). Thus, it is paramount to better understand ways of encouraging park visitation and physical activity and social interaction in the park among adults to enhance health and well-being.

Previous studies have demonstrated associations between certain park characteristics and adults’ park use and park-based physical activity (Kaczynski *et al*. 2014). For example, a recent study conducted among adults in Singapore found that the provision of water features, forested areas, and aesthetics was positively associated with park visit duration, and open green space was positively associated with park-based physical activity (Petrunoff *et al*. 2022). A systematic review found that the presence of lighting and paths/trails was positively associated with adults’ park-based physical activity (Zhang *et al*. 2018), and two studies not included in the review reported positive associations between good condition and cleanliness and observed park-based physical activity among adults (Hamilton *et al*. 2017), and between larger park size, presence of water features, lighting along paths, car parking, bike racks, and walking/cycling paths and self-reported physical activity in green spaces (Schipperijn *et al*. 2013). These findings are congruent with qualitative studies, which have shown that park facilities (e.g. walking and cycling paths), aesthetics, and social interaction with others can support adults’ park-based physical activity (Groshong *et al*. 2017, Veitch *et al*. 2022b), and physical features (e.g. facilities, size), social factors (e.g. bad social behaviour and social support) and individual factors (e.g. time, weather) can influence park choice for active use (Aliyas 2020). Natural experiment studies have also demonstrated that upgrading or adding new park features may increase adults’ park visits and active park use (Veitch *et al*. 2018, Cohen *et al*. 2019, Poppe *et al*. 2023, Paudel *et al*. 2024), and are a cost-effective strategy for increasing park-based physical activity (Lal *et al*. 2019). However, little is known about the importance of individual park features such as paths, trees, and lighting for encouraging active park visits.

Further, parks are also important for providing opportunities for social interaction with others, which may contribute to social connectedness and social cohesion, and enhance overall well-being (Hari and Kujala 2009, Maas *et al*. 2009, Jose *et al*. 2012, Clarke *et al*. 2023). For example, the presence of green spaces has been shown to increase social interaction among local residents and foster social cohesion (Bergefurt *et al*. 2019). However, little is known about which specific park features may encourage adults’ social interaction in parks. This is important as these park features may differ from those that facilitate park visitation and park-based physical activity (Rivera *et al*. 2021). A recent review that examined factors that impact social cohesion in urban greenspaces including parks, forests, and gardens found that reducing crime, improving maintenance and accessibility, and having physical space and amenities for social gatherings for diverse demographic groups were the most important considerations (Clarke *et al*. 2023). A recent qualitative study conducted in Australia, which used walk-along interviews to explore adults’ perceptions of important park features, found that seating, tables, and barbecue/picnic areas were important for encouraging social interaction in parks (Veitch *et al*. 2022b).

Previous studies have shown gender differences in preferred park features among children (Veitch *et al*. 2021b), adolescents (Veitch *et al*. 2016, Rivera *et al*. 2021), and older adults (Veitch *et al*. 2022a), but this requires further investigation among adults as it is unknown whether the importance of features for encouraging active and social park visits differs for males and females. Frequency of park visitation may also influence which park features are important for encouraging park visits. However, to our knowledge, this has not been examined among adults (Rivera *et al*. 2021). A better understanding of whether preferred park features vary according to gender and frequency of park visitation is important to ensure that parks are designed to meet the needs of various user groups.

The aim of this study was to describe the perceived importance of a selection of park features for encouraging park visitation, park-based physical activity, and park-based social interaction among adults (19–64 years) by asking them to rate images/written descriptions of park features. Differences in ratings according to gender and frequency of park visitation were also examined. This cross-sectional survey study used a photographic rating methodology to determine the perceived importance that adults place on specific park features for encouraging park use and to describe the importance of park features for all three park outcomes (visitation, physical activity, and social interaction).

## METHODS

In January 2021, adults aged 19–64 years living in metropolitan Melbourne, Australia, were invited to complete an online survey. A survey panel company emailed invitations to potential participants (*n* = 1861) via their database of adults aged 19–64 years living in metropolitan Melbourne. A total of 277 adults responded; however, participants were excluded if they did not meet the age requirement (*n* = 7) or did not consent to participate (*n* = 1). After incomplete surveys were removed (*n* = 37), a sample of 232 adults with complete surveys remained. Due to outdated information in the database, some participants were from other states (*n* = 13) or regional areas in Victoria (*n* = 1).

Qualtrics was used to create and administer the online survey, which contained digital images/written descriptions of 43 different park features. Photographic studies are an innovative way to examine adults’ perceptions of park features as the characteristics can be viewed without participants having to actually visit parks and this method also makes it clear to both the participant and researcher which feature is under consideration. The park features were selected based on features that adults perceived as being important for park visitation, park-based physical activity, and social interaction in a previous qualitative walk-along study (Veitch *et al*. 2022b). The chosen features were consistent with the social-ecological model (Sallis *et al*. 2006) and varied in nature, including both physical infrastructure (e.g. paths, drink taps, toilets), environmental qualities (e.g. trees, gardens), individual considerations (facilities for different age groups), and social factors (other people in the park, sense of safety from strangers) (see Supplementary File 1).

**Figure 1.**
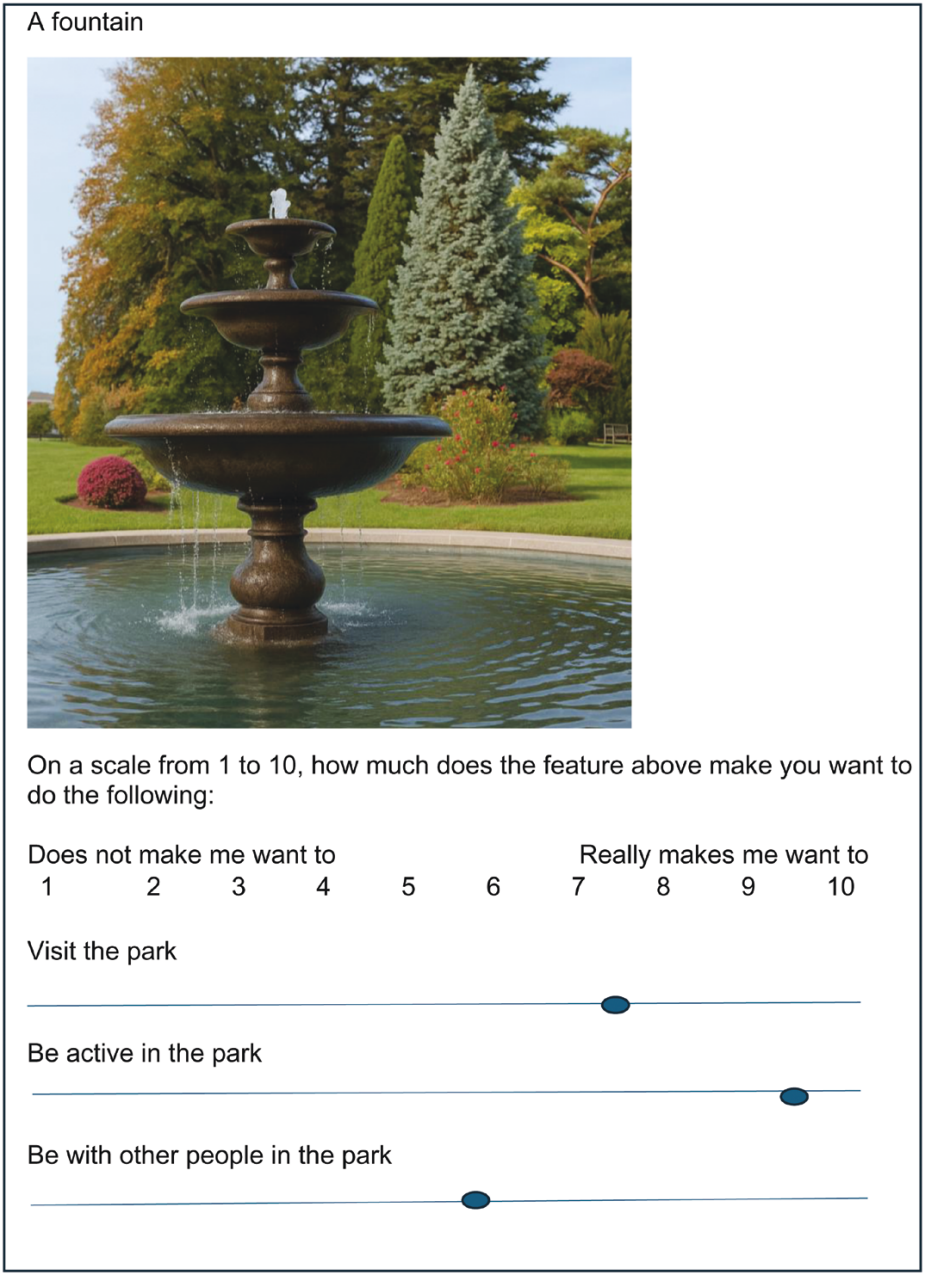
Example of rating task.

Participants were asked to rate each feature on a scale of 1 to 10 for each of the three park-use behaviours [i.e. how much does this feature make you want to (1) visit the park, (2) be active in the park, and (3) be with other people in the park; 1 = does not make me want to, 10 = really makes me want to—see Fig. 1]. The features were presented individually and in the same order for each participant. Where possible, images were standardized on certain factors, such as being taken at eye level. Images were chosen to highlight the specific feature being assessed, while minimizing other visual elements that could distract from the intended feature, and where possible, they depicted real-world park settings with similar lighting and backgrounds. Most features were portrayed with one image; however, two images were shown in some instances to more clearly depict the feature. For example, for ‘netball and basketball courts’, an image of both types of courts were shown and for ‘birdlife’, an image of birds in a tree and ducks on a pond were included. Six features were difficult to depict via an image (i.e. good maintenance and cleanliness, a peaceful and relaxed setting, sense of safety from strangers and undesirable people, facilities suitable for children of different ages, park is a large size, and a variety of activities) and these features were described with words. Images were either original photographs taken in parks around Melbourne, or free-to-use stock images.

Demographic questions were included in the survey to gather information on the participants’ age, gender, highest level of education, country of birth, number of children or grandchildren, dog ownership, and number of years living at current address. Participants were also asked questions about their general park usage over the past 3 months. This included frequency of visitation dichotomized to infrequent park visitor (< once per week) and frequent park visitor (≥ once per week), length of visit (< 30 min, 30–60 min, > 1 – <2h, ≥2h), activities undertaken when visiting (mostly sitting or lying down, mostly light activities, mostly moderate activities, mostly vigorous activities), usual method of travel to the park (walked, jogged, cycled, car, public transport, others), and who they spent time with at the park (alone without dog, friend(s), child(ren), grandchild(ren), dog(s), another adult from family, organized group, other) (Table 1). The survey took on average 35 min to complete and participants were offered a $15 gift voucher as compensation for their time.

**Table 1.** Demographics and park visitation characteristics

	Overall *n* = 232	Male *n* = 110	Female *n* = 122
Age, mean (SD)	42.2 (13.4)	47.9 (13.6)	45.5 (13.1)
Gender, female (%)	52.6		
Education level (%)			
Did not complete high school	6.5	7.3	5.7
Year 12/trade/certificate	33.2	32.7	33.6
University or tertiary qualification	60.3	60.0	60.7
Country of birth (%)			
Australia	75.4	74.6	76.2
Other	24.6	25.4	23.8
Dog ownership (%)	39.2	39.1	39.3
At least one child ≤12 years old (%)	20.3	20.9	21.3
At least one child 13–18 years old (%)	18.6	18.2	19.7
At least one grandchild (%)	15.1	15.5	14.7
Years living in current address, mean (SD)	10.1 (10.1)	10.5 (9.1)	9.6 (11.0)
Usual frequency of park visits in past 3 months (%)			
≤once per week	33.6	31.8	35.2
≥once per week	53.5	60.0	47.5
Not visited in the past 3 months	12.9	8.2	17.2
Usual duration of park visits in past 3 months (%)^a^			
<30 min	14.7	16.4	13.1
30–60 min	49.1	48.2	50.0
>1– <2 h	17.7	20.0	15.6
2 or more hours	5.6	7.3	4.1
Method of travel to park (%)^a^^,^^b^			
Walked	78.7	83.2	74.3
Jogged	13.9	13.9	13.9
Cycled	13.9	18.8	8.9
Car	39.6	39.6	39.6
Public transport	5.5	8.9	1.9
Other	1.0	1.0	1.0
Usual accompaniment during park visits in past 3 months (%)^a^^,^^b^			
Alone without dog	32.7	63.4	71.3
Friend(s)	35.1	31.6	38.6
Child(ren)	29.7	23.7	35.6
Grandchild(ren)	9.9	10.9	8.9
Dog(s)	27.2	23.7	30.7
Another adult from family	50.0	53.5	46.5
Organized group	1.9	1.9	1.9
Other	3.0	3.0	3.0
Usual activity levels during park visits in past 3 months (%)^a^			
Mostly sitting or lying down	7.0	9.9	4.0
Mostly light activities	53.2	44.5	62.0
Mostly moderate activities	37.3	43.5	31.0
Mostly vigorous activities	2.5	1.9	3.0

^a^Only presented to respondents who indicated that they had visited a park in the past 3 months.

^b^Multiple responses were possible.

### Data analysis

Data were downloaded from Qualtrics, and descriptive statistics and rating scores were calculated using Stata/SE version 17.0. For each feature, mean rating scores (SD) were calculated for the whole sample and separately for males and females and for frequent park visitors and infrequent park visitors for each of the three park use behaviours (park visitation, park-based physical activity, social interaction). Independent sample *t*-tests compared differences in mean rating scores between males and females and between frequent and infrequent park visitors for each feature and condition (*P* < .05). Mean rating scores for each subgroup are presented in Supplementary File 2. A rating from 1 to 43 (1 representing highest mean rating score) was calculated for each of the three park use behaviours for the whole sample, for males and females, and for frequent and infrequent park visitors.

## RESULTS

The mean age was 42.2 years (SD = 13.4), and 53% of participants were female. More than half (60%) had a university or tertiary qualification, and most (75%) were born in Australia. More than half (53%) reported visiting parks at least once/week in the past 3 months, and 49% reported spending between 30 and 60 min in the park during their visit. Most participants (78%) reported walking as their usual mode of travel to the park and ‘light activities’ was the most commonly reported activity performed during park visits (Table 1).

### Park features most important for visitation

The ratings of features for encouraging park visitation are shown in Table 2. Overall, ‘good maintenance and cleanliness’ was the highest-rated feature for encouraging adults to visit the park (highest-rated for males and second highest for females). ‘Trees’ and a ‘peaceful and relaxed setting’ were the next two most important features, followed by ‘sense of safety’ and a ‘water feature’. The lowest-rated features included: climbing equipment, dog facilities, bike lock station, playground for younger children, trampolines, sports wall to play different sports against, netball or basketball courts, and skate park.

**Table 2.** Adults ratings of park features for encouraging park visitation

Feature	Overall mean score (SD)	Overall rating	Male rating	Female rating	Gender difference in mean rating scores (*P*-value^*^)	Frequent visitor rating	In-frequent visitor rating	Frequent/infrequent difference in mean rating scores (*P*-value^*^)
Good maintenance/cleanliness	8.20 (1.87)	1	1	2	.379	1	1.5	**.041**
Trees	8.09 (1.89)	2	2	3	.194	2	4	**.012**
Peaceful and relaxed setting	8.03 (1.92)	3	3	4	.356	3	3	.105
Sense of safety from strangers	8.02 (2.04)	4	6	1	**.021**	5	1.5	.551
Water feature (pond/lake)	7.90 (2.06)	5	4	6	.758	6	5	.195
Birdlife	7.82 (2.12)	6	5	7	.891	4	7	**.033**
Gardens	7.74 (2.12)	7	10	5	.095	9	6	.282
Creek	7.71 (2.15)	8	8	8	.526	7	8	**.020**
Natural environment/native plants/gardens	7.63 (2.17)	9	9	9	.732	10	9	.072
Trees that provide shade	7.60 (2.05)	10	7	10	.826	8	11	**.008**
Quiet, secluded spots	7.42 (2.31)	11	13	11	.743	12	10	.309
Large grassy open space	7.35 (2.03)	12.5	11.5	13	.540	11	13	**.013**
Park is a large size	7.35 (2.25)	12.5	11.5	12	.723	13	12	.378
Clean toilets	7.04 (2.51)	14.5	14	18	.148	14	17	**.010**
BBQ or picnic area	7.04 (2.36)	14.5	15	14	.555	15	14	.155
Variety of amenities/things to do	6.96 (2.60)	16	16	15	.459	16	15	.350
Fountain	6.78 (2.48)	17	20.5	16	.445	20	16	.500
Concrete, smooth, sealed path	6.78 (2.39)	18	19	17	.634	18.5	18	.239
Café, coffee cart	6.70 (2.51)	19.5	23	19	.381	18.5	20	.107
Gravel/natural walking path	6.70 (2.34)	19.5	17	22	.450	17	21.5	**.036**
Tables and chairs	6.66 (2.40)	21	22	20	.677	22	19	.193
Traditional park seats	6.63 (2.42)	22	20.5	23	.804	21	21.5	.115
Built shelter	6.50 (2.36)	23	18	27	.073	25	23	.146
Lighting	6.49 (2.46)	24	24	25	.634	24	24	.068
Interactive features (light up when touched)	6.34 (3.05)	25	28	24	.226	26.5	25	.147
Herb/vegetable garden	6.34 (2.74)	26	29	21	.108	26.5	25	.101
Park open and clearly visible from the street	6.23 (2.43)	27	25	28	.713	28	28	**.030**
Car parking	6.21 (2.58)	28	30	26	.179	30	27	.227
Drink taps	6.06 (2.56)	29	26	30	.351	23	32	**<.0001**
Other people in park	5.91 (2.59)	30	27	33	.212	29	31	**.001**
Adventure playground for older children	5.87 (3.11)	31	31	29	.641	31	29	.061
Facilities for different age groups	5.77 (3.04)	32	32	31	.788	32	30	**.043**
Places for parents to sit and watch children	5.63 (2.99)	33	34	32	.541	34	35	**.023**
Outdoor fitness equipment	5.63 (2.95)	34	33	37	.808	35	34	**.033**
Shade over playground	5.55 (2.96)	35	35	34	.519	37	33	**.089**
Climbing equipment	5.50 (3.02)	36	36	36	.596	36	36	**.040**
Dog facilities	5.46 (2.98)	37	38	35	.440	33	38	**.001**
Bike lock station	5.18 (2.81)	38	37	39	.439	38	37	**.028**
Playground for younger children	5.04 (3.04)	39	39	40	.728	39	39	**.021**
Trampolines	4.91 (3.00)	40	41	38	.242	40	40	**.042**
Sports wall	4.81 (2.78)	41	40	41	.968	41	41	**.018**
Netball/basketball courts	4.46 (2.92)	42	42	42	.336	42	42	.196
Skate park	3.65 (2.69)	43	43	43	.915	43	43	**.009**

^*^Significant difference in mean scores between groups was set at *P* < 0.05 (bolded) using independent sample *t*-tests.

There was one significant difference in mean rating scores between genders. ‘Sense of safety from strangers and undesirable people’ was rated first for females and sixth for males (mean score for females = 8.33 vs male = 7.73, *P* = .021). For frequent/infrequent visitors, there were significant differences in mean rating scores for 22 of the 43 features (Table 2). For example, ‘trees’ were rated second for frequent visitors and fourth for infrequent visitors (mean score for frequent = 7.78 vs infrequent = 6.75, *P* = .012).

### Park features most important for park-based physical activity

Consistent with the findings for park visitation, ‘good maintenance and cleanliness’, and ‘trees’ were the top two-rated features overall for encouraging park-based physical activity (Table 3). ‘Sense of safety from strangers and undesirable people’ was the third highest-rated feature followed by ‘large size’ and a ‘water feature’. Two features that appeared in the top 10 for encouraging park-based physical activity that were not in the top ten for visitation included the ‘park being a large size’ (rated fourth) and a ‘concrete, smooth, sealed path’ (rated seventh).

**Table 3.** Adults ratings of park features for encouraging park-based physical activity

Feature	Overall mean score (SD)	Overall rating	Male rating	Female rating	Gender difference in mean rating scores (*P*-value^*^)	Frequent visitor rating	In-frequent visitor rating	Frequent/infrequent difference in mean rating scores (*P*-value^*^)
Good maintenance/cleanliness	7.34 (2.38)	1	2	1	.548	1	2	**.023**
Trees	7.28 (2.31)	2	1	3	.437	2	3	**.015**
Sense of safety from strangers	7.25 (2.48)	3	3	2	.377	3	1	.148
Park is a large size	6.91 (2.52)	4	5	4	.866	5	4	.085
Water feature (pond/lake)	6.83 (2.59)	5	4	8	.273	4	7	**.016**
Gardens	6.70 (2.54)	6	11	5	.473	12	5	.420
Concrete, smooth, sealed path	6.69 (2.48)	7	12	6	.413	8	6	.198
Creek	6.68 (2.56)	8	6	10	.305	6	10	**.008**
Natural environment/native plants/gardens	6.58 (2.58)	9	14	9	.720	7	11	**.033**
Birdlife	6.54 (2.50)	10	9	11	.650	14	9	.100
Variety of amenities/things to do	6.53 (2.72)	11	15.5	7	.420	15	8	.389
Large grassy open space	6.49 (2.31)	12	7	15	**.047**	=8	12	**.006**
Trees that provide shade	6.42 (2.36)	13.5	13	12	.405	13	13	**.007**
Gravel/natural walking path	6.42 (2.50)	13.5	10	13	.244	11	15	**.004**
Peaceful and relaxed setting	6.27 (2.65)	15	17	14	.778	18	14	.102
Clean toilets	6.26 (2.76)	16	8	20	**.034**	=8	20	**<.001**
BBQ or picnic area	6.23 (2.51)	17	15.5	17	.329	17	17	**.013**
Fountain	6.09 (2.57)	18	21	16	.886	20	16	.118
Lighting	6.00 (2.56)	19	24	18	.705	19	18.5	**.033**
Drink taps	6.00 (2.55)	20	19	19	.603	16	28	**<.001**
Quiet, secluded spots	5.91 (2.64)	21	20	23.5	.213	23.5	18.5	.123
Built shelter	5.87 (2.43)	22	18	27.5	.069	21	22	**.016**
Park open and clearly visible from the street	5.83 (2.48)	23	22	23.5	.346	26	21	**.043**
Outdoor fitness equipment	5.82 (3.04)	24	25	21	.783	23.5	24	.068
Tables and chairs	5.74 (2.57)	25	23	30.5	.184	28	23	.112
Café, coffee cart	5.69 (2.70)	26	29	25	.966	25	31	**.006**
Car parking	5.69 (2.66)	27	27	27.5	.634	29	25	.121
Traditional park seats	5.66 (2.56)	28	28	30.5	.475	30	26	.100
Adventure playground for older children	5.65 (3.09)	29	30	22	.782	27	30	.067
Herb/vegetable garden	5.63 (2.73)	30	31	26	.820	31.5	27	.094
Other people in park	5.60 (2.67)	31	26	35	.231	22	35	**.001**
Facilities for different age groups	5.48 (3.01)	32	33	29	.736	31.5	33.5	**.021**
Interactive features (e.g. light when touched)	5.44 (2.98)	33	35	32	.770	38	29	.394
Climbing equipment	5.35 (3.03)	34.5	34	36	.856	35	33.5	.095
Bike lock station	5.35 (2.81)	34.5	32	37	.731	33	36	**.014**
Shade over the playground	5.33 (2.93)	36	36	34	.723	37	32	.127
Dog facilities	5.24 (2.95)	37	38	33	.446	34	38	**.003**
Places for parents to sit and watch children	5.23 (2.93)	38	37	38	.737	36	37	**.027**
Sports wall	5.08 (2.87)	39	39	40	.968	39	39	**.018**
Trampolines	4.98 (2.99)	40	42	39	.269	40	40	**.038**
Playground for younger children	4.87 (2.99)	41	40	41	.663	41	41	**.025**
Netball/basketball courts	4.66 (2.94)	42	41	42	.530	42	42	.059
Skate park	3.58 (2.58)	43	43	43	.414	43	43	.088

^*^Significant difference in mean scores between groups was set at *P* < 0.05 (bolded) using independent sample *t*-tests.

There were two significant differences in mean rating scores between genders. ‘Large grassy open space’ was rated seventh for males and fifteenth for females (mean score for males = 6.81 vs females = 6.22, *P* = .047); and clean toilets were rated eighth for males and twentieth for females (mean score for males = 6.68 vs females = 5.92, *P* = .034). For frequent/infrequent visitors, there were significant differences in mean rating scores for 23 of the 43 features (Table 3). For example, a ‘water feature’ was rated fourth for frequent visitors and seventh for infrequent visitors (mean score for frequent = 7.21 vs infrequent = 6.40, *P* = .016).

### Park features most important for social interaction

The three most highly rated park features for encouraging social interaction were the same as those for encouraging physical activity ‘good maintenance and cleanliness’, ‘trees’, and ‘sense of safety from strangers and undesirable people’. The fourth and fifth highest-rated features were ‘gardens’ and a ‘bbq or picnic area’ (Table 4). The lowest-rated features were similar to those for park visitation.

**Table 4.** Adults ratings of park features for encouraging park-based social interaction

Feature	Overall mean score (SD)	Overall rating	Male rating	Female rating	Gender difference in mean rating scores (*P*-value^*^)	Frequent visitor rating	In-frequent visitor rating	Frequent/infrequent difference in mean rating scores (*P*-value^*^)
Good maintenance/cleanliness	7.42 (2.58)	1	1	1	.171	1	1	**.003**
Trees	7.29 (2.62)	2	2	2	.140	2	5	**.002**
Sense of safety from strangers	7.17 (2.70)	3	6	4	.076	6	2	.079
Gardens	7.16 (2.59)	4.5	7	3	**.037**	5	4	**.030**
BBQ or picnic area	7.16 (2.54)	4.5	3	5	.157	7	3	.065
Water feature (pond/lake)	7.13 (2.64)	6	4.5	6	.206	3	6	**.008**
Peaceful and relaxed setting	7.02 (2.80)	7	4.5	8	.501	4	9	**.003**
Park is a large size	6.88 (2.77)	8	8	11	.439	14	7	.094
Birdlife	6.88 (2.62)	9.5	10	10	.331	8	11.5	**.006**
Café, coffee cart	6.88 (2.64)	9.5	15.5	7	.069	12	8	**.037**
Quiet, secluded spots	6.85 (2.85)	11	13.5	9	.151	9	13	**.008**
Creek	6.84 (2.73)	12	9	14	.598	10	14	**.006**
Trees that provide shade	6.82 (2.48)	13	12	13	.317	13	10	**.020**
Natural environment/native plants/gardens	6.77 (2.73)	14	11	15	.473	11	15	**.006**
Variety of amenities/things to do	6.76 (2.86)	15	15.5	12	.232	17	11.5	.059
Tables and chairs	6.65 (2.66)	16	18	16	.332	15	17	**.002**
Large grassy open space	6.56 (2.51)	17	17	17.5	.722	18	16	**.013**
Built shelter	6.51 (2.67)	18	13.5	19	.797	19	18	**.017**
Clean toilets	6.38 (2.93)	19	19	21	.899	16	24	**<.001**
Concrete, smooth, sealed path	6.35 (2.73)	20	23	17.5	.258	22	19	**.048**
Fountain	6.31 (2.70)	21	22	20	.416	23	20	**.037**
Traditional park seats	6.31 (2.63)	22	21	22	.506	20	21	**.002**
Gravel/natural walking path	6.22 (2.75)	23	20	24	.938	21	23	**.003**
Interactive features (e.g. lights when touched)	6.15 (3.15)	24	26	23	.195	25	22	.065
Lighting	6.00 (2.82)	25	27	26	.383	26	25.5	**.018**
Car parking	5.95 (2.80)	26	28	25	.305	29	25.5	**.031**
Other people in park	5.93 (2.77)	27	24	30.5	.645	24	31	**.001**
Adventure playground for older children	5.91 (3.13)	28	29	27	.376	28	27	**.024**
Park open and clearly visible from the street	5.90 (2.59)	29	25	29	.870	27	28	**.001**
Herb/vegetable garden	5.82 (2.91)	30	31	28	.281	30	29	**.012**
Facilities suitable different age groups	5.75 (3.12)	31	30	32	.791	31	30	**.024**
Places for parents to sit and watch children	5.63 (3.04)	32	35	30.5	.257	32	33	**.004**
Shade over the playground	5.59 (3.08)	33	33	33	.462	33	32	**.017**
Climbing equipment	5.47 (3.08)	34	34	34	.755	35	34	**.020**
Drink taps	5.41 (2.66)	35	32	35	.702	34	36	**.001**
Outdoor fitness equipment	5.30 (3.10)	36	36	36	.748	37	35	.077
Playground for younger children	5.11 (3.14)	37	38	37.5	.793	39	37	**.016**
Bike lock station	5.11 (2.86)	38	37	39	.965	38	38	**.006**
Dog facilities	5.06 (2.98)	39	39	37.5	.621	36	41	**.001**
Trampolines	5.00 (3.01)	40	41	40	.515	40	39	**.034**
Sports wall	4.93 (2.88)	41	40	41	.781	41	40	**.009**
Netball/basketball courts	4.66 (3.02)	42	42	42	.715	42	42	.067
Skate park	3.67 (2.67)	43	43	43	.536	43	43	**.036**

^*^Significant difference in mean scores between groups was set at *P* < 0.05 (bolded) using independent sample *t*-test.

There was one significant difference in mean rating scores between genders. ‘Gardens’ were rated third for females and seventh for males (mean score for females = 7.51 vs males = 6.80, *P* = .037). For frequent and infrequent visitors, there were significant differences in mean rating scores for 36 of the 43 features (Table 4). For example, ‘gardens’ were rated fifth for frequent visitors and fourth for infrequent visitors (mean score for frequent = 7.51 vs infrequent = 6.77, *P* = .030).

## DISCUSSION

This study described how adults (19–64 years) perceived the significance of 43 pre-determined park features for encouraging park visitation, park-based physical activity, and social interaction in parks and examined differences in ratings according to gender and frequency of park visitation. Limited research has explored the perspectives of adults regarding the importance of individual park features for physical activity and social interaction within the park.

Overall, good maintenance and cleanliness was the highest-rated feature for all three park use behaviours (park visitation, park-based physical activity, social interaction). This is consistent with a previous cross-sectional survey conducted among adults in Melbourne, which found that good park maintenance was the most important feature out of 20 park features for engaging in physical activity (Costigan *et al*. 2017). An observational study of six parks in Canada also showed a positive association between well-maintained and clean parks and levels of observed physical activity among park visitors (Hamilton *et al*. 2017). Additionally, a review of qualitative studies found that good park maintenance was important for encouraging park use (McCormack *et al*. 2010) and, in a recent qualitative walk-along study, improved park maintenance was frequently suggested by Australian adults as being critical for increasing park visitation (Veitch *et al*. 2022b). This highlights to park managers the importance of prioritizing good maintenance and cleanliness.

Natural elements, such as trees, water features, and gardens, were also perceived as important for all three park use behaviours. Other natural elements, such as birdlife, creeks, and native plants, were rated highly for both park visitation and park-based physical activity. This is unsurprising as previous research has shown that natural elements within parks were positively associated with park visit duration and park-based physical activity among Asian adults (Petrunoff *et al*. 2022). Further, natural elements can contribute to the aesthetic appeal of the park and create a peaceful and relaxed setting, which was another highly rated feature in our study for encouraging park visitation and social interaction. Australian older adults have also been found to prefer parks with natural elements (Veitch *et al*. 2020, 2022a). This further emphasizes the importance of incorporating natural elements into park design as it can entice different age groups to visit.

A sense of safety from strangers and undesirable people was important for encouraging park visitation and physical activity, with a greater importance placed on this feature by females than males. Recent qualitative walk-along interviews conducted among Australian adults highlighted the importance of safety for park use and enjoyment, particularly personal safety related to the presence of undesirable or suspicious people. These concerns were mentioned as a major issue for female participants in a previous study in Melbourne (Veitch *et al*. 2022b). While addressing safety concerns related to strangers and undesirable individuals is a complex societal issue, the provision of lighting and other surveillance technology may help to improve perceived safety of parks for adult visitors (Groshong *et al*. 2020). Interestingly, safety did not emerge as a key concern in previous research using the same methodology examining important park features with children (Veitch *et al*. 2021b), adolescents (Rivera *et al*. 2021), and older adults (Veitch *et al*. 2022a). However, previous research with children and adolescents has identified that safety concerns influence use of parks and public open spaces (Babey *et al*. 2008, Pawlowski *et al*. 2019).

BBQ or picnic areas, and cafés or coffee carts were particularly important for social interaction among adults in our study. Incorporating BBQs, picnic areas, and cafes in park design is likely to be appealing to a range of age groups, as they also have been found to be highly valued for encouraging social interaction among adolescents and older adults (Rivera *et al*. 2021, Veitch *et al*. 2022a). BBQs, picnic areas, and cafes can provide opportunities for people to gather and socialize which may encourage people to spend more time in the park. A cross-sectional study conducted among adults in Mexico suggested that to improve well-being of park visitors, it is important to prioritize features that promote social interaction (Ayala-Azcárraga *et al*. 2019). A natural experiment in Belgium found no effect of a park refurbishment, that included the addition of picnic tables but not BBQ’s or cafes/coffee carts, on social interaction among park visitors (Poppe *et al*. 2023). Future natural experiments should examine the impact of including these park features in park refurbishments on social interaction among different age groups.

Of the features examined, eight were found to be among the 10 lowest-rated features for all three park-use behaviours: climbing equipment, dog facilities, bike lock station, playground for younger children, trampolines, sports wall to play different sports against, netball or basketball courts, and a skate park. Most of these features are more appealing to different age groups. For example, in a study conducted in Australia, netball or basketball courts were among the most important features for physical activity and social interaction among adolescents (Rivera *et al*. 2021), and climbing structures were rated highly by children for encouraging active and social park visits (Veitch *et al*. 2021a). Future research may wish to examine how adventure playgrounds or playgrounds for younger children are rated among parents with different-aged children. This emphasizes that park design should consider the needs and preferences of different age groups. Whilst our study identified few differences in mean rating scores between genders, a large number of significant differences in mean rating scores were observed between frequent and infrequent park visitors for all three park-use behaviours. This highlights the importance of considering the needs of both frequent and infrequent park visitors when designing parks as it is critical to create parks that attract infrequent visitors to visit more often.

### Strengths and limitations

Strengths include the examination of the importance of specific park features for three distinct park-use behaviours: park visitation; park-based physical activity; and social interaction. This is important as it provides an understanding of park features that may promote park-based physical activity and social interaction as well as park visitation. A further strength is the examination of preferred features by gender and frequency of park visitation. Although previous studies have used digital images to examine preferences for park features among children (Veitch *et al*. 2021a), adolescents (Veitch *et al*. 2016, Van Hecke *et al*. 2018, Rivera *et al*. 2021), and older adults (Veitch *et al*. 2022a), to our knowledge, this is the first study to use this methodology among adults. Digital images have been reported to be a novel way of examining park features among adults (Nordh *et al*. 2009, Nordh 2012) and are beneficial as participants do not have to imagine the features or visit parks in person to view the features, the features rated are consistent for all participants and therefore less open to interpretation than written descriptions (Nordh 2012), and responses to coloured, digital images have been shown to be valid when compared to on-site features (Stamps 1993). However, we acknowledge that there may be some limitations as the actual images used may have influenced the ratings given (i.e. type of gardens, fitness equipment), the weather shown in the images varied slightly across images (i.e. sunny/grey sky), the greenery in the background of the images may have influenced the rating, and static images may be perceived differently to on-site features viewed in real life. Furthermore, 6 of the 43 features could not be clearly depicted as an image (e.g. size, safety, and maintenance) as they were characteristics/qualities of parks (as opposed to physical features, such as a walking path) and thus were described in words and 14 features had two images. It is important for future research to explore ratings of park features during real-world park visits and compare the findings to those reported in this study. In addition, although the 43 features were selected based on qualitative research with adults (Veitch *et al*. 2022b), it is possible that other park features may also be important and an order effect may have influenced the results as all the images were shown in the same order for all participants, and test-retest reliability was not explored. Finally, the sample available through the survey panel company who participated in this study may not be representative of the broader population. Future studies should consider including adults who live in regional and rural areas and in other countries where the neighbourhood environment and parks may differ.

## CONCLUSION

There is significant potential for parks to confer multiple health and social benefits for the adult population by increasing park visitation and enhancing physical activity and social interaction. The most valued features perceived to support diverse park-based behaviours among adults included good maintenance and cleanliness, trees, a peaceful and relaxed setting, and a sense of safety from strangers and undesirable people. Few differences in mean rating scores were observed between genders; however, many differences in mean rating scores were observed for frequent and infrequent park visitors. This highlights the importance of considering varied user groups when planning new urban parks. Future studies should examine the relative importance of features that were identified in this study to ensure park design supports physical, mental, and social health and well-being.

## Supplementary Material

daaf063_suppl_Supplementary_Files_1

daaf063_suppl_Supplementary_Files_2

## Notes

AUTHOR CONTRIBUTIONS

J.V.: conceptualization, funding acquisition, data collection, formal analysis, interpretation, writing—original draft preparation, and writing—review & editing; E.R., V.L.: interpretation, writing—original draft preparation, and writing—review & editing; N.B.: data collection, writing—original draft preparation, and writing—review & editing; C.P., B.D., A.T.: conceptualization, interpretation, and writing—review & editing. All authors provided critical intellectual feedback on drafts and read and approved the final manuscript version.

CONFLICT OF INTEREST
