# Supplementary material for: Examining park features that encourage physical activity and social interaction among adults
Source: Health Promot Int. 2025 Jun 2;40(3):daaf063. doi: 10.1093/heapro/daaf063 (PMC12128928; doi:10.1093/heapro/daaf063)
Supplement: daaf063_suppl_Supplementary_Files_1 [file daaf063_suppl_supplementary_files_1.docx]

**Supplementary File 1 – Summary of Features**

| **Summary of 43 Features** | **Image** |  |
| --- | --- | --- |
| Playground: |  |  |
| - Playground for younger children | 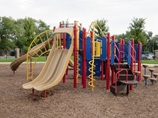 |  |
| - Adventure playground for older children | 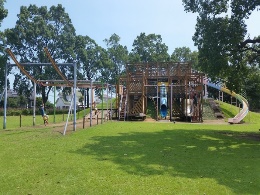 | 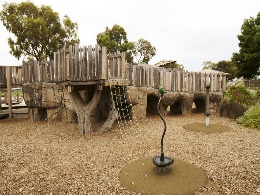 |
| - Shade over the playground | 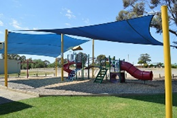 |  |
| - Climbing equipment | 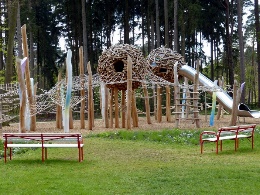 | 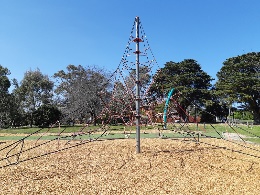 |
| - Park open and clearly visible from the street | 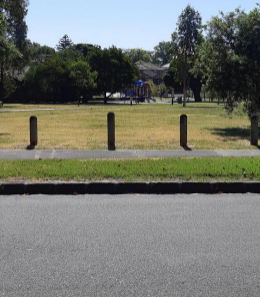 |  |
| - Skate park | 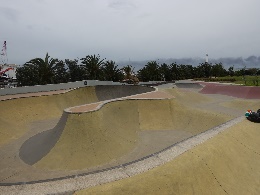 |  |
| - Trampolines | 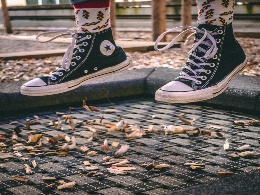 | 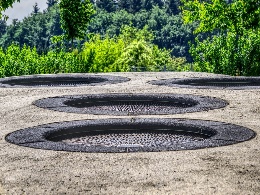 |
| - Places for parents to sit and watch children | 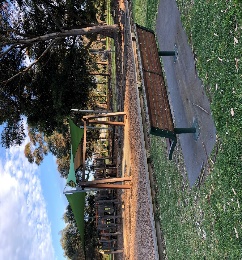 | 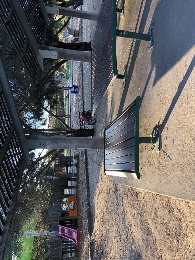 |
| Nature: |  |  |
| - Trees | 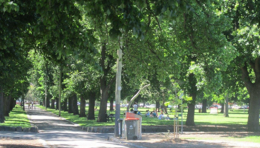 |  |
| - Gardens | 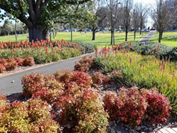 | 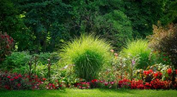 |
| - Large grassy open space | 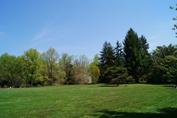 |  |
| - Birdlife | 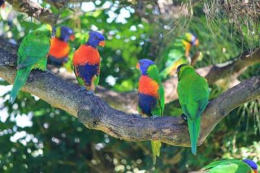 | 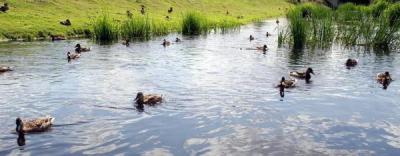 |
| - Trees that provide shade | 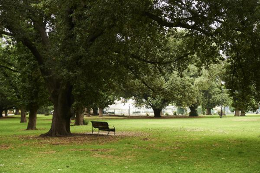 |  |
| - A creek | 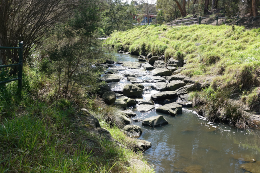 |  |
| - Water feature like a pond or lake | 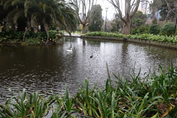 |  |
| - Herb or vegetable garden | 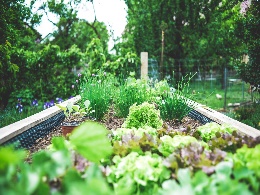 |  |
| - Natural environment/native plants and gardens | 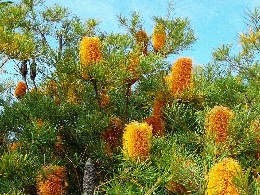 |  |
| Paths: |  |  |
| - Concrete, smooth, sealed path | 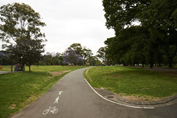 | 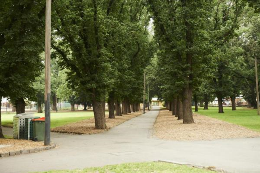 |
| - Gravel/natural walking path | 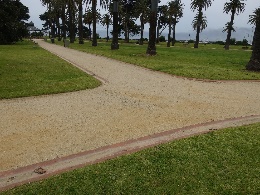 | 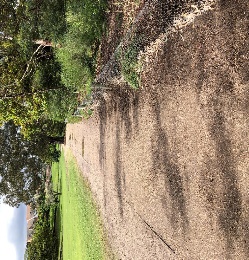 |
| Signage: |  |  |
| - Dog facilities | 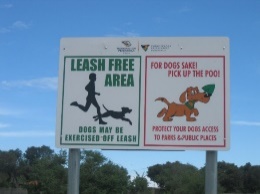 | 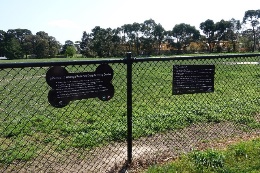 |
| Seating: |  |  |
| - Traditional park seats | 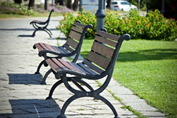 |  |
| - Table and chairs | 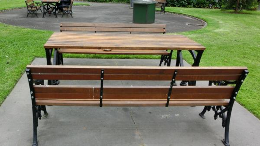 |  |
| Netball or basketball courts | 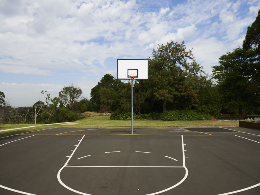 | 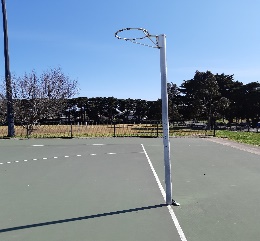 |
| Car parking | 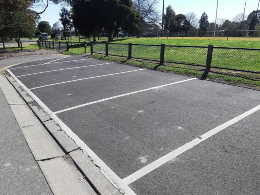 |  |
| Other people in the park | 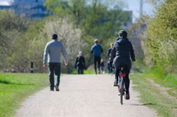 | 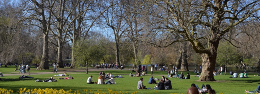 |
| A fountain | 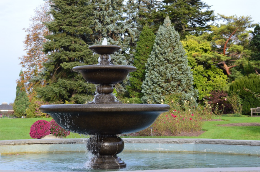 |  |
| Sports wall to play different sports against | 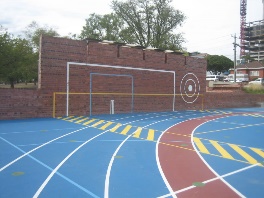 |  |
| Outdoor fitness equipment | 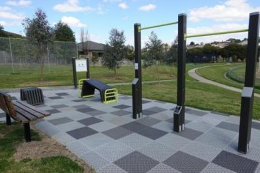 | 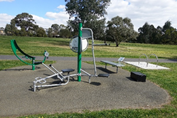 |
| Interactive features that light up with colours or make sounds when you touch them | 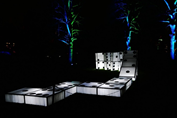 | 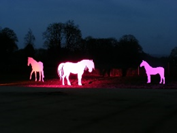 |
| Built shelter | 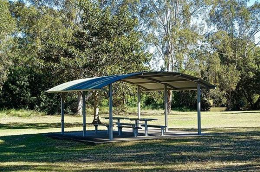 |  |
| Lighting | 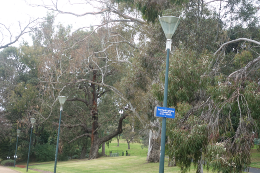 |  |
| Quiet, secluded spots | 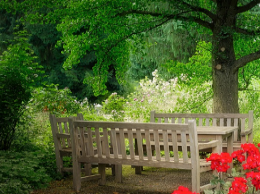 |  |
| Café, coffee cart | 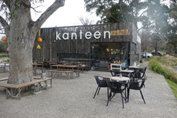 |  |
| BBQ or picnic area | 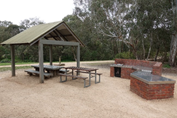 |  |
| Drink taps | 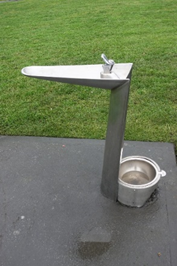 |  |
| Clean toilets | 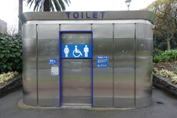 |  |
| Bike lock station | 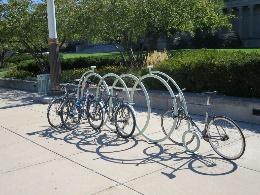 | 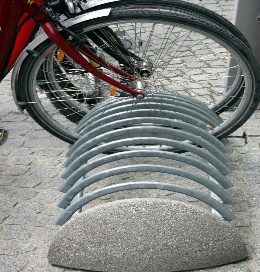 |
| A peaceful and relaxed setting | No picture |  |
| Good maintenance and cleanliness | No picture |  |
| Sense of safety from strangers and undesirable people | No picture |  |
| Facilities suitable for children of different ages | No picture |  |
| Park is a large size | No picture |  |
| Variety of activities/amenities/things to do | No picture |  |
