# Supplementary material for: Examining park features that encourage physical activity and social interaction among adults
Source: Health Promot Int. 2025 Jun 2;40(3):daaf063. doi: 10.1093/heapro/daaf063 (PMC12128928; doi:10.1093/heapro/daaf063)
Supplement: daaf063_suppl_Supplementary_Files_2 [file daaf063_suppl_supplementary_files_2.docx]

**Supplementary File 2**

**Mean scores (SD) of park features for encouraging park visitation**

| **Feature** | **Overall mean score (SD)** | **Male**  **mean score (SD)** | **Female mean score (SD)** | **Frequent visitor mean score (SD)** | **Infrequent visitor mean score (SD)** |
| --- | --- | --- | --- | --- | --- |
| Good maintenance & cleanliness | 8.20 (1.87) | 8.11 (1.77) | 8.32 (1.88) | 8.44 (1.53) | 7.94 (2.16) |
| Trees | 8.09 (1.89) | 7.94 (1.73) | 8.26 (1.93) | 8.39 (1.48) | 7.76 (2.22) |
| Peaceful and relaxed setting | 8.03 (1.92) | 7.91 (1.77) | 8.13 (1.96) | 8.22 (1.68) | 7.81 (2.14) |
| Sense of safety from strangers and undesirable people | 8.02 (2.04) | 7.73 (1.98) | 8.33 (1.98) | 8.09 (1.77) | 7.94 (2.32) |
| Water feature like a pond or lake | 7.90 (2.06) | 7.87 (2.05) | 7.95 (2.01) | 8.07 (1.80) | 7.71 (2.30) |
| Birdlife | 7.82 (2.12) | 7.82 (1.76) | 7.86 (2.35) | 8.11 (1.68) | 7.51 (2.50) |
| Gardens | 7.74 (2.12) | 7.52 (2.16) | 7.97 (2.00) | 7.89 (1.91) | 7.58 (2.24) |
| Creek | 7.71 (2.15) | 7.63 (2.05) | 7.81 (2.16) | 8.02 (1.80) | 7.36 (2.44) |
| Natural environment/native plants and gardens | 7.63 (2.17) | 7.60 (2.08) | 7.70 (2.19) | 7.87 (1.91) | 7.35 (2.40) |
| Trees that provide shade | 7.60 (2.05) | 7.64 (1.82) | 7.58 (2.18) | 7.93 (1.65) | 7.24 (2.38) |
| Quiet, secluded spots | 7.42 (2.31) | 7.37 (2.17) | 7.47 (2.40) | 7.57 (2.21) | 7.25 (2.42) |
| Large grassy open space | 7.35 (2.03) | 7.44 (1.80) | 7.28 (2.14) | 7.66 (1.74) | 7.01 (2.26) |
| Park is a large size | 7.35 (2.25) | 7.44 (2.03) | 7.34 (2.30) | 7.48 (2.14) | 7.22 (2.37) |
| Clean toilets | 7.04 (2.51) | 7.32 (2.12) | 6.85 (2.75) | 7.44 (2.16) | 6.60 (2.80) |
| BBQ or picnic area | 7.04 (2.36) | 6.94 (2.11) | 7.13 (2.53) | 7.25 (2.27) | 6.81 (2.44) |
| Variety of activities/  amenities/things to do | 6.96 (2.60) | 6.86 (2.43) | 7.11 (2.68) | 7.11 (2.57) | 6.79 (2.63) |
| Fountain | 6.78 (2.48) | 6.67 (2.34) | 6.92 (2.56) | 6.89 (2.42) | 6.66 (2.55) |
| Concrete, smooth, sealed path | 6.78 (2.39) | 6.72 (2.35) | 6.87 (2.42) | 6.95 (2.34) | 6.58 (2.44) |
| Café, coffee cart | 6.70 (2.51) | 6.54 (2.37) | 6.82 (2.59) | 6.95 (2.30) | 6.43 (2.70) |
| Gravel/natural path | 6.70 (2.34) | 6.84 (2.21) | 6.61 (2.43) | 7.01 (2.21) | 6.36 (2.44) |
| Tables and chairs | 6.66 (2.40) | 6.59 (2.24) | 6.72 (2.52) | 6.84 (2.32) | 6.47 (2.50) |
| Traditional park seats | 6.63 (2.42) | 6.67 (2.20) | 6.59 (2.56) | 6.86 (2.33) | 6.36 (2.43) |
| Built shelter | 6.50 (2.36) | 6.79 (2.03) | 6.24 (2.57) | 6.71 (2.15) | 6.26 (2.56) |
| Lighting | 6.49 (2.46) | 6.41 (2.36) | 6.56 (2.51) | 6.77 (2.44) | 6.18 (2.46) |
| Interactive features that light up with colours or make sounds when you touch them | 6.34 (3.05) | 6.08 (2.87) | 6.57 (3.15) | 6.61 (2.87) | 6.04 (3.22) |
| Herb or vegetable garden | 6.34 (2.74) | 6.05 (2.67) | 6.63 (2.77) | 6.61 (2.61) | 6.03 (2.87) |
| Park open and clearly visible from the street | 6.23 (2.43) | 6.29 (2.16) | 6.18 (2.66) | 6.56 (2.40) | 5.86 (2.43) |
| Car parking | 6.21 (2.58) | 5.97 (2.58) | 6.43 (2.55) | 6.40 (2.50) | 5.99 (2.65) |
| Drink taps | 6.06 (2.56) | 6.25 (2.53) | 5.93 (2.55) | 6.79 (2.32) | 5.25 (2.59) |
| Other people in the park | 5.91 (2.59) | 6.12 (2.42) | 5.70 (2.70) | 6.44 (2.44) | 5.32 (2.63) |
| Adventure playground for older children | 5.87 (3.11) | 5.78 (3.10) | 5.97 (3.14) | 6.23 (2.94) | 5.46 (3.26) |
| Facilities suitable for children of different ages | 5.77 (3.04) | 5.72 (2.91) | 5.82 (3.18) | 6.16 (2.82) | 5.35 (3.23) |
| Places for parents to sit and watch children | 5.63 (2.99) | 5.50 (2.87) | 5.74 (3.12) | 6.06 (2.81) | 5.16 (3.13) |
| Outdoor fitness equipment | 5.63 (2.95) | 5.67 (3.03) | 5.57 (2.90) | 6.02 (2.88) | 5.19 (2.98) |
| Shade over playground | 5.55 (2.96) | 5.42 (2.74) | 5.67 (3.16) | 5.86 (2.78) | 5.20 (3.11) |
| Climbing equipment | 5.50 (3.02) | 5.41 (2.95) | 5.62 (3.10) | 5.89 (2.88) | 5.07 (3.13) |
| Dog facilities | 5.46 (2.98) | 5.34 (2.97) | 5.64 (2.97) | 6.14 (2.90) | 4.71 (2.91) |
| Bike lock station | 5.18 (2.81) | 5.38 (2.82) | 5.09 (2.80) | 5.57 (2.80) | 4.75 (2.76) |
| Playground for younger children | 5.04 (3.04) | 5.12 (2.84) | 4.98 (3.21) | 5.48 (2.94) | 4.55 (3.08) |
| Trampolines | 4.91 (3.00) | 4.68 (2.96) | 5.14 (3.04) | 5.30 (2.97) | 4.49 (2.98) |
| Sports wall to play different sports against | 4.81 (2.78) | 4.84 (2.70) | 4.82 (2.87) | 5.22 (2.78) | 4.36 (2.73) |
| Netball/basketball courts | 4.46 (2.92) | 4.66 (2.85) | 4.29 (2.96) | 4.70 (2.84) | 4.20 (2.99) |
| Skate park | 3.65 (2.69) | 3.68 (2.67) | 3.64 (2.71) | 4.08 (2.83) | 3.17 (2.45) |

**Mean scores (SD) of park features for encouraging physical activity in the park**

| **Feature** | **Overall mean**  **score (SD)** | **Male mean score (SD)** | **Female mean score (SD)** | **Frequent visitor mean score (SD)** | **Infrequent visitor mean score (SD)** |
| --- | --- | --- | --- | --- | --- |
| Good maintenance & cleanliness | 7.34 (2.38) | 7.26 (2.24) | 7.45 (2.46) | 7.68 (2.19) | 6.97 (2.52) |
| Trees | 7.28 (2.31) | 7.42 (2.07) | 7.19 (2.45) | 7.63 (2.03) | 6.90 (2.53) |
| Sense of safety from strangers and undesirable people | 7.25 (2.48) | 7.12 (2.24) | 7.41 (2.64) | 7.48 (2.18) | 7.00 (2.77) |
| Park is a large size | 6.91 (2.52) | 6.97 (2.41) | 6.92 (2.54) | 7.18 (2.48) | 6.62 (2.56) |
| Water feature like a pond or lake | 6.83 (2.59) | 7.05 (2.26) | 6.68 (2.84) | 7.21 (2.35) | 6.40 (2.79) |
| Gardens | 6.70 (2.54) | 6.60 (2.34) | 6.84 (2.68) | 6.83 (2.45) | 6.55 (2.64) |
| Concrete, smooth, sealed path | 6.69 (2.48) | 6.57 (2.43) | 6.83 (2.53) | 6.89 (2.42) | 6.46 (2.54) |
| Creek | 6.68 (2.56) | 6.87 (2.32) | 6.53 (2.73) | 7.10 (2.43) | 6.22 (2.62) |
| Natural environment/native plants and gardens | 6.58 (2.58) | 6.54 (2.38) | 6.66 (2.72) | 6.92 (2.42) | 6.20 (2.79) |
| Birdlife | 6.54 (2.50) | 6.63 (2.10) | 6.48 (2.80) | 6.80 (2.25) | 6.25 (2.74) |
| Variety of activities/ amenities/things to do | 6.53 (2.72) | 6.41 (2.54) | 6.69 (2.82) | 6.67 (2.60) | 6.36 (2.85) |
| Large grassy open space | 6.49 (2.31) | 6.81 (1.93) | 6.22 (2.53) | 6.89 (2.08) | 6.05 (2.48) |
| Trees that provide shade | 6.42 (2.36) | 6.56 (2.07) | 6.30 (2.56) | 6.81 (2.04) | 5.98 (2.61) |
| Gravel/natural path | 6.42 (2.50) | 6.62 (2.33) | 6.24 (2.62) | 6.86 (2.28) | 5.93 (2.64) |
| Peaceful and relaxed setting | 6.27 (2.65) | 6.33 (2.46) | 6.23 (2.75) | 6.54 (2.46) | 5.97 (2.82) |
| Clean toilets | 6.26 (2.76) | 6.68 (2.35) | 5.92 (3.01) | 6.89 (2.42) | 5.56 (2.95) |
| BBQ or picnic area | 6.23 (2.51) | 6.41 (2.24) | 6.08 (2.72) | 6.61 (2.39) | 5.80 (2.58) |
| Fountain | 6.09 (2.57) | 6.08 (2.39) | 6.13 (2.73) | 6.34 (2.45) | 5.81 (2.67) |
| Lighting | 6.00 (2.56) | 5.92 (2.37) | 6.04 (2.68) | 6.34 (2.54) | 5.63 (2.54) |
| Drink taps | 6.00 (2.55) | 6.13 (2.63) | 5.96 (2.45) | 6.65 (2.38) | 5.28 (2.55) |
| Quiet, secluded spots | 5.91 (2.64) | 6.12 (2.38) | 5.69 (2.83) | 6.16 (2.59) | 5.63 (2.68) |
| Built shelter | 5.87 (2.43) | 6.20 (2.13) | 5.62 (2.65) | 6.23 (2.21) | 5.46 (2.60) |
| Park open and clearly visible from the street | 5.83 (2.48) | 6.00 (2.20) | 5.69 (2.72) | 6.14 (2.42) | 5.48 (2.51) |
| Outdoor fitness equipment | 5.82 (3.04) | 5.88 (3.08) | 5.77 (3.03) | 6.16 (2.97) | 5.44 (3.09) |
| Tables and chairs | 5.74 (2.57) | 5.98 (2.31) | 5.53 (2.77) | 5.99 (2.51) | 5.45 (2.51) |
| Café, coffee cart | 5.69 (2.70) | 5.70 (2.48) | 5.68 (2.89) | 6.16 (2.55) | 5.18 (2.78) |
| Car parking | 5.69 (2.66) | 5.78 (2.65) | 5.62 (2.67) | 5.94 (2.56) | 5.40 (2.74) |
| Traditional park seats | 5.66 (2.56) | 5.77 (2.41) | 5.53 (2.68) | 5.92 (2.50) | 5.36 (2.61) |
| Adventure playground for older children | 5.65 (3.09) | 5.60 (2.98) | 5.72 (3.21) | 6.00 (2.94) | 5.25 (3.22) |
| Herb or vegetable garden | 5.63 (2.73) | 5.58 (2.62) | 5.67 (2.84) | 5.91 (2.67) | 5.31 (2.78) |
| Other people in the park | 5.60 (2.67) | 5.80 (2.50) | 5.38 (2.78) | 6.19 (2.52) | 4.95 (2.70) |
| Facilities suitable for children of different ages | 5.48 (3.01) | 5.42 (2.80) | 5.56 (3.20) | 5.91 (2.83) | 5.00 (3.14) |
| Interactive features that light up with colours or make sounds when you touch them | 5.44 (2.98) | 5.38 (2.83) | 5.49 (3.10) | 5.60 (2.85) | 5.26 (3.13) |
| Climbing equipment | 5.35 (3.03) | 5.41 (2.93) | 5.33 (3.13) | 5.66 (2.87) | 5.00 (3.17) |
| Bike lock station | 5.35 (2.81) | 5.45 (2.78) | 5.32 (2.88) | 5.78 (2.74) | 4.87 (2.83) |
| Shade over playground | 5.33 (2.93) | 5.25 (2.68) | 5.39 (3.16) | 5.61 (2.81) | 5.02 (3.04) |
| Dog facilities | 5.24 (2.95) | 5.11 (2.98) | 5.41 (2.92) | 5.77 (2.97) | 4.65 (2.82) |
| Places for parents to sit and watch children | 5.23 (2.93) | 5.17 (2.80) | 5.30 (3.07) | 5.63 (2.84) | 4.78 (2.98) |
| Sports wall to play different sports against | 5.08 (2.87) | 5.08 (2.79) | 5.10 (2.98) | 5.50 (2.86) | 4.61 (2.82) |
| Trampolines | 4.98 (2.99) | 4.75 (2.96) | 5.19 (3.03) | 5.37 (2.89) | 4.55 (3.04) |
| Playground for younger children | 4.87 (2.99) | 4.97 (2.81) | 4.80 (3.15) | 5.29 (2.94) | 4.41 (2.99) |
| Netball/basketball courts | 4.66 (2.94) | 4.79 (2.90) | 4.55 (2.97) | 5.00 (2.80) | 4.27 (3.04) |
| Skate park | 3.58 (2.58) | 3.74 (2.60) | 3.46 (2.56) | 3.85 (2.63) | 3.27 (2.50) |

**Mean scores (SD) of park features for encouraging social interaction in the park**

| **Feature** | **Overall mean**  **score (SD)** | **Male mean score (SD)** | **Female mean**  **score (SD)** | **Frequent visitor mean score (SD)** | **Infrequent visitor mean score (SD)** |
| --- | --- | --- | --- | --- | --- |
| Good maintenance & cleanliness | 7.42 (2.58) | 7.19 (2.58) | 7.65 (2.54) | 7.89 (2.10) | 6.90 (2.95) |
| Trees | 7.29 (2.62) | 7.03 (2.65) | 7.53 (2.55) | 7.78 (2.23) | 6.75 (2.92) |
| Sense of safety from strangers and undesirable people | 7.17 (2.70) | 6.85 (2.67) | 7.47 (2.67) | 7.47 (2.34) | 6.85 (3.02) |
| Gardens | 7.16 (2.59) | 6.80 (2.62) | 7.51 (2.50) | 7.51 (2.33) | 6.77 (2.82) |
| BBQ or picnic area | 7.16 (2.54) | 6.92 (2.55) | 7.38 (2.48) | 7.45 (2.19) | 6.84 (2.85) |
| Water feature like a pond or lake | 7.13 (2.64) | 6.91 (2.68) | 7.34 (2.56) | 7.57 (2.28) | 6.65 (2.92) |
| Peaceful and relaxed setting | 7.02 (2.80) | 6.91 (2.73) | 7.15 (2.78) | 7.52 (2.46) | 6.46 (3.04) |
| Park is a large size | 6.88 (2.77) | 6.75 (2.75) | 7.03 (2.73) | 7.17 (2.47) | 6.56 (3.06) |
| Birdlife | 6.88 (2.62) | 6.71 (2.44) | 7.04 (2.75) | 7.32 (2.20) | 6.39 (2.95) |
| Café, coffee cart | 6.88 (2.64) | 6.55 (2.55) | 7.18 (2.68) | 7.22 (2.27) | 6.50 (2.97) |
| Quiet, secluded spots | 6.85 (2.85) | 6.56 (2.89) | 7.09 (2.78) | 7.31 (2.48) | 6.34 (3.15) |
| Creek | 6.84 (2.73) | 6.74 (2.75) | 6.93 (2.70) | 7.30 (2.37) | 6.33 (3.02) |
| Trees that provide shade | 6.82 (2.48) | 6.65 (2.46) | 6.97 (2.46) | 7.18 (2.20) | 6.43 (2.71) |
| Natural environment/native plants and gardens | 6.77 (2.73) | 6.66 (2.73) | 6.92 (2.70) | 7.23 (2.37) | 6.25 (3.00) |
| Variety of activities/  amenities/things to do | 6.76 (2.86) | 6.55 (2.85) | 6.99 (2.80) | 7.10 (2.59) | 6.39 (3.10) |
| Tables and chairs | 6.65 (2.66) | 6.46 (2.62) | 6.80 (2.67) | 7.14 (2.31) | 6.10 (2.91) |
| Large grassy open space | 6.56 (2.51) | 6.50 (2.53) | 6.62 (2.47) | 6.95 (2.26) | 6.14 (2.70) |
| Built shelter | 6.51 (2.67) | 6.56 (2.63) | 6.47 (2.70) | 6.90 (2.48) | 6.07 (2.80) |
| Clean toilets | 6.38 (2.93) | 6.37 (2.78) | 6.42 (3.04) | 7.13 (2.37) | 5.55 (3.26) |
| Concrete, smooth, sealed path | 6.35 (2.73) | 6.11 (2.76) | 6.62 (2.65) | 6.69 (2.47) | 5.98 (2.95) |
| Fountain | 6.31 (2.70) | 6.17 (2.59) | 6.46 (2.79) | 6.66 (2.47) | 5.93 (2.89) |
| Traditional park seats | 6.31 (2.63) | 6.18 (2.59) | 6.41 (2.64) | 6.81 (2.37) | 5.75 (2.81) |
| Gravel/natural path | 6.22 (2.75) | 6.19 (2.74) | 6.22 (2.75) | 6.71 (2.45) | 5.66 (2.96) |
| Interactive features that light up with colours or make sounds when you touch them | 6.15 (3.15) | 5.86 (3.06) | 6.39 (3.17) | 6.51 (2.93) | 5.75 (3.34) |
| Lighting | 6.00 (2.82) | 5.80 (2.80) | 6.13 (2.82) | 6.41 (2.74) | 5.54 (2.86) |
| Car parking | 5.95 (2.80) | 5.76 (2.78) | 6.14 (2.81) | 6.33 (2.74) | 5.54 (2.81) |
| Other people in the park | 5.93 (2.77) | 6.01 (2.79) | 5.84 (2.75) | 6.61 (2.44) | 5.16 (2.92) |
| Adventure playground for older children | 5.91 (3.13) | 5.73 (3.07) | 6.09 (3.19) | 6.35 (2.89) | 5.43 (3.32) |
| Park open and clearly visible from the street | 5.90 (2.59) | 5.88 (2.47) | 5.93 (2.71) | 6.40 (2.43) | 5.35 (2.66) |
| Herb or vegetable garden | 5.82 (2.91) | 5.59 (2.83) | 6.01 (2.99) | 6.27 (2.78) | 5.32 (2.99) |
| Facilities suitable for children of different ages | 5.75 (3.12) | 5.71 (3.03) | 5.82 (3.23) | 6.19 (2.92) | 5.26 (3.28) |
| Places for parents to sit and watch children | 5.63 (3.04) | 5.39 (2.96) | 5.84 (3.12) | 6.16 (2.86) | 5.04 (1.43) |
| Shade over playground | 5.59 (3.08) | 5.43 (2.90) | 5.73 (3.26) | 6.04 (2.97) | 5.08 (1.13) |
| Climbing equipment | 5.47 (3.08) | 5.42 (2.99) | 5.54 (3.18) | 5.91 (2.89) | 4.97 (3.22) |
| Drink taps | 5.41 (2.66) | 5.50 (2.69) | 5.37 (2.61) | 6.02 (2.57) | 4.75 (2.60) |
| Outdoor fitness equipment | 5.30 (3.10) | 5.34 (3.08) | 5.21 (3.12) | 5.64 (3.06) | 4.92 (3.11) |
| Playground for younger children | 5.11 (3.14) | 5.07 (2.98) | 5.18 (3.30) | 5.58 (2.99) | 4.59 (3.23) |
| Bike lock station | 5.11 (2.86) | 5.14 (2.88) | 5.13 (2.87) | 5.59 (2.80) | 4.57 (2.84) |
| Dog facilities | 5.06 (2.98) | 4.98 (3.03) | 5.18 (2.94) | 5.75 (2.95) | 4.29 (2.83) |
| Trampolines | 5.00 (3.01) | 4.85 (3.04) | 5.11 (3.01) | 5.40 (2.98) | 4.56 (3.00) |
| Sports wall to play different sports against | 4.93 (2.88) | 4.88 (2.82) | 4.98 (2.97) | 5.39 (2.86) | 4.41 (2.83) |
| Netball/basketball courts | 4.66 (3.02) | 4.75 (3.04) | 4.60 (3.01) | 5.01 (2.93) | 4.28 (3.08) |
| Skate park | 3.67 (2.67) | 3.79 (2.62) | 3.58 (2.71) | 4.02 (2.73) | 3.28 (2.55) |

|  |  |  |
| --- | --- | --- |
